# Seroprevalence and Risk Factors of Crimean–Congo Hemorrhagic Fever Exposure in Wild and Domestic Animals in Benin

**DOI:** 10.3390/v17030387

**Published:** 2025-03-08

**Authors:** Roland Eric Yessinou, Souaïbou Farougou, James Olukayode Olopade, Daniel Oladimeji Oluwayelu, Anise Happi, Christian Happi, Martin Groschup

**Affiliations:** 1 Communicable Diseases Research Unit, Department of Production and Animal Health, University of Abomey-Calavi, Abomey Calavi 229, Benin; eric.yessinou@gmail.com (R.E.Y.); s.farougou@gmail.com (S.F.); 2 Department of Veterinary Anatomy, Faculty of Veterinary Medicine, University of Ibadan, Ibadan 200284, Nigeria; 3 Humboldt Research Hub for Zoonotic Arboviral Diseases, Faculty of Veterinary Medicine, University of Ibadan, Ibadan 200284, Nigeria; ogloryus@yahoo.com; 4 Department of Veterinary Microbiology, Faculty of Veterinary Medicine, University of Ibadan, Ibadan 200284, Nigeria; 5 African Center of Excellence for Genomics of Infectious Diseases (ACEGID), Redeemer’s University, Oshogbo 232102, Nigeria; anisehappi@yahoo.com (A.H.); happic@run.edu.ng (C.H.); 6 Friedrich-Loeffler-Institut, Institute of Novel and Emerging Infectious Diseases, Südufer 10, 17493 Greifswald, Insel Riems, Germany; martin.groschup@fli.de

**Keywords:** viral surveillance, crimean-congo hemorrhagic fever virus, Benin, West Africa

## Abstract

Crimean–Congo hemorrhagic fever (CCHF) is a tick-borne zoonotic viral disease prevalent in Africa. While infection is asymptomatic in animals, it can cause severe illness with hemorrhagic manifestations and high mortality rates in humans. This study aimed to determine the seroprevalence and potential risk factors of CCHF in wild (rodents, birds) and domestic (cattle, horses) animals in Benin. A cross-sectional study was carried out from 2022 to 2024 with the assistance of cattle breeders, hunters, farmers and bushmeat sellers in 15 districts found in three agroecological zones in the country. A total of 366 serum samples were analyzed, comprising 254 collected from wild animals and 112 from domestic animals. Among the wild animals tested, 1.18% (95% CI: 0.31–3.70; n = 3) were seropositive for antibodies against CCHF virus (CCHFV). The seroprevalence rates were 3.7% (95% CI: 0.19–20.89) in squirrels, 5.88% (95% CI: 0.31–30.76) in hares and 1.19% (95% CI: 0.06–7.38) in giant rats. In domestic animals, anti-CCHFV antibodies were detected in 38 of the 112 samples, resulting in an overall seroprevalence of 33.93% (95% CI: 25.42–43.56). Specifically, antibodies were identified in 34 out of 81 cattle (41.98%, 95% CI: 31.26–53.46) and 4 out of 24 horses (16.67%, 95% CI: 5.48–38.19). No positive samples were reported in pigeons. This study provides the first seroprevalence data on CCHF in wild and domestic animals in Benin. It highlights the risk and epidemiological dynamics of the disease and underscores the need for further investigations into tick vectors and human populations.

## 1. Introduction

Viral hemorrhagic fevers constitute a significant public health concern in many tropical and subtropical regions, with the emergence and re-emergence of vector-borne viral diseases playing a prominent role among zoonotic diseases [1,2]. Crimean–Congo hemorrhagic fever virus (CCHFV), a tick-borne virus, has a wide geographical distribution in Africa [3]. While ticks of the genus *Hyalomma* are the primary vectors of CCHFV, the virus has also been reported in *Amblyomma* and *Rhipicephalus* species worldwide [4]. Tick dynamics in endemic regions appear to play a role in the emergence of CCHFV and may influence the risk of tick-borne pathogen transmission to humans [5]. CCHFV persists throughout a tick’s lifespan, providing continuous opportunities to infect domestic and wild animals as well as humans, while maintaining the virus in the environment [6]. However, infections with CCHFV can also occur through contact with blood, body fluids, or infectious tissues. CCHF is characterized by fever, headache, tremors, nausea, abdominal pain, vomiting and arthralgia in humans [7]. These clinical symptoms can often be mistaken for malaria due to limited diagnostic and surveillance capacities in developing countries [8]. Furthermore, CCHF infection is considered to be a significant threat to livestock farmers, veterinarians and slaughterhouse workers due to increased exposure to tick bites and animal body fluids. The transmission of CCHFV infections has also been reported among healthcare workers [9].

Evidence of CCHF infection has been documented in birds, horses, donkeys, goats, cattle, sheep and pigs in various regions of Europe, Asia and Africa [10]. However, CCHFV may spread beyond its current geographic range through the introduction of ticks via migratory birds, transhumance, livestock trade, agricultural land expansion and habitat fragmentation [11,12]. Environmental and climatic changes and wild animals are also factors contributing to the emergence and spread of CCHFV [13,14]. Additionally, close proximity between humans and domestic or wild animals, coupled with poor biosecurity measures, increases the risk of zoonotic viral infections [15]. CCHF outbreaks have severe consequences on human health due to their infectious capacity, significant morbidity and mortality rate and challenges related to its diagnosis, treatment and prevention [16].

In West Africa, CCHFV has been reported in rural areas of countries such as Mauritania, Mali, Gambia, Senegal, Niger, Burkina Faso and Nigeria [3,17,18,19]. Evidence of viral infections reported in ruminants, ticks and humans suggests a natural circulation of the virus in this sub-region [3]. In Benin, however, there is a paucity of information on the presence of CCFHV despite serological and/or molecular evidence of its presence in neighboring countries as mentioned earlier. The simultaneous presence of competent vectors and favorable ecological factors suggests a potential increased risk of CCHFV emergence in the country. The current study is the first of its kind in Benin, aimed at obtaining epidemiological data on the seroprevalence of CCHFV infection and assessing associated risk factors in domestic animals. The study provides for the first time critical information on the presence of CCHFV and highlights the potential endemicity of the virus in the country.

## 2. Materials and Methods

### 2.1. Study Area

The study area included northern and southern Benin, encompassing 15 districts distributed across the 3 agroecological zones of West Atacora zone IV, Central Benin Cotton zone V and Northern Benin cotton zone II. Northern Benin is characterized through a semi-arid Sudanian climate beyond latitude 10°N with a unimodal rainfall pattern (900 to 1100 mm of annual precipitation) and two distinct seasons: one dry and one rainy. The region features dense dry forests, open forests, savannah and grassland reserves [20]. In contrast, southern Benin has a subequatorial climate with a bimodal rainfall pattern with four seasons: two rainy seasons and two dry seasons. The region is known for its fertile soil, although ecological conditions have degraded over time. Annual rainfall reaches up to 1500 mm, and the vegetation consists of forest–savannah mosaics, fallow land and cultivated fields. Both southern and northern Benin are home to a rich diversity of wild animal species. Domestic cattle are mainly raised under the sedentary, nomadic and semi-extensive farming systems [21,22]. The main phenotypes of cattle raised include Lagune, Somba, Pabli and Borgou alongside several breeds of zebu introduced through transhumance and crossbreeding programs [23]. In addition, the government of Benin imported the N’dama, Girolando and Azawak to strengthen cattle production [24,25].

### 2.2. Animal Sample Collection

A cross-sectional study was conducted from 2022 to 2024 among cattle breeders, hunters, farmers and bushmeat sellers in the districts of Aplahoué, Allada, Adjarra, Kpomassè, Djidja, Adja-Ouèrè, Tanguiéta, Banikoara, urban and rural Djougou, Gogounou, Matéri, Natitingou, Parakou and Pobè. Sampling sites were identified randomly with the assistance of para-veterinarians in the study districts. The domestic animals were sampled in the farms and horse stables and were not closely associated with the wild animals. Blood samples from wild and domestic animals were collected into appropriately labeled dry serum separator tubes. The blood samples were allowed to clot and then centrifuged at 25,000× *g* for 10 min. The separated serum samples were refrigerated at 4 °C, before being transported to the URMAT laboratory, where they were stored in a −20 °C freezer until testing.

### 2.3. Serological Analysis

Serum samples were tested using the CCHF dual-antigen multi-species enzyme-linked immunosorbent assay (ELISA) kit (IDScreen; IDvet, Grables, France), following the manufacturer’s instructions. Briefly, 30 μL of each test or control sample was pre-diluted with 50 μL of the kit diluent in a separately available 96-well microtiter dilution or processing plate (Thermo Scientific, Waltham, MA, USA) before the mixture was transferred to the antigen-coated plate and incubated for 45 min at 25 °C. After a wash procedure, 50 μL of conjugate was added to each test well, followed by incubation for 30 min at 25 °C. A second wash procedure was then performed and 100 μL of substrate was added, followed by incubation for 15 min at 25 °C before stopping the reaction. An optical density value for each test or control sample was determined through spectrophotometry at 450 nm. Subsequently, the determination of the seropositivity or negativity threshold for CCHF was performed according to the kit criteria.

### 2.4. Statistical Analysis

Data were entered and validated using a Microsoft™ Excel spreadsheet. Seroprevalence rates followed by 95% confidence intervals were determined using the prop.test function in R. Odds ratios and their corresponding confidence intervals were determined using a Fisher exact test after organizing the data into contingency tables. All statistical analyses were performed using R.4.3.2 software. The results were considered statistically significant if the *p*-value < 0.05.

## 3. Results

**Figure 1:**
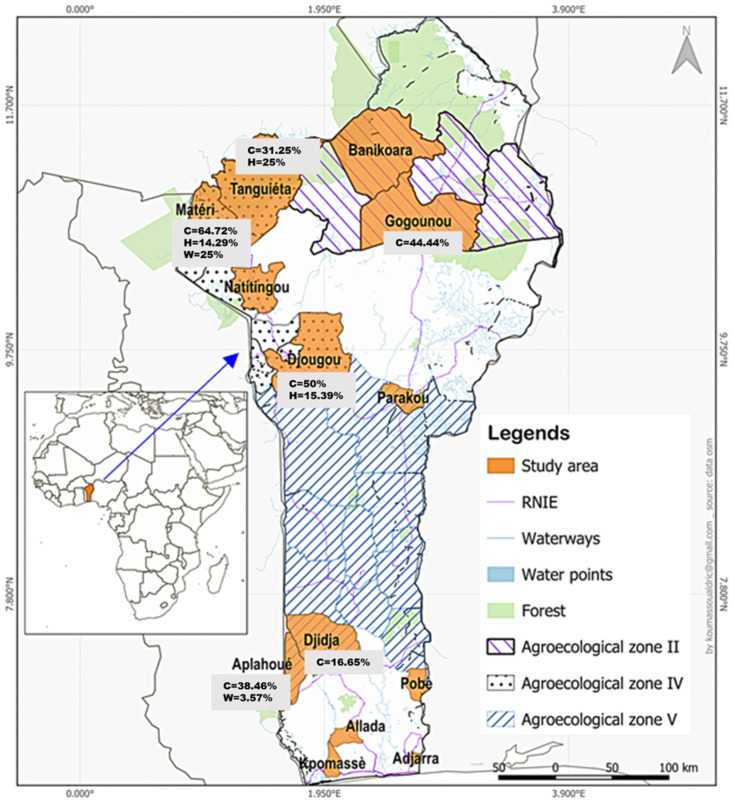
A map of the study sites in districts in south and north Benin. C, H and W indicates % seroprevalence of CCHFV in the study sites. The seroprevalence is 0% where no data is shown.

A total of 366 serum samples were analyzed, including 254 collected from wild animals and 112 from domestic animals. Among the wild animals, 1.18% (95% CI: 0.31–3.70; n = 3) were seropositive for antibodies against CCHFV. Seroprevalences of 3.7% (95% CI: 0.19–20.89) and 5.88% (95% CI: 0.31–30.76) were reported for squirrels and hares, respectively, but in giant rats the seroprevalence was 1.19% (95% CI: 0.06–7.38). While the seroprevalence in hares showed a trend toward significance, no significant differences were obtained in giant rats or squirrels. All other wild animal samples tested were negative for CCHFV antibodies. Regarding the study sites (Figure 1), a seroprevalence of 25% (95% CI: 1.32–78.06) was observed in Matéri, but the seroprevalence reported in the district of Aplahoué was 3.57% (95% CI: 0.62–13.38). The odds ratio for anti-CCHFV seropositivity was 8.33 (*p* < 0.04, 95% CI: 0.12–209.41) (Table 1).

Of the 112 domestic animal samples tested, anti-CCHFV antibodies were detected in 38 domestic animals, representing a seroprevalence of 33.93% (95% CI: 25.42–43.56). Anti-CCHFV antibodies were detected in 34/81 cattle (41.98%, 95% CI: 31.26–53.46) and 4/24 horses (16.67%, 95% CI: 5.48–38.19) tested, but no positive samples were reported in pigeons. The seroprevalence obtained for cattle was higher than that for horses and was statistically significant (OR = 0.28; *p* = 0.013; 95% CI: 0.06–0.94). Overall, CCHFV antibodies in cattle were identified in six districts, with the highest seroprevalence detected in Matéri (64.71%, 95% CI: 38.62–84.74). Moreover, the odds of anti-CCHFV seropositivity were high in Djidja (OR = 2.21; *p* = 0.197; 95% CI: 0.28–28.21) compared to other districts. In horses, seropositive samples were found only in the districts of Tanguiéta, Djougou and Matéri. The highest seropositivity was recorded in Tanguiéta (25%; 95% CI: 1.32–78.06) while similar seropositivity rates were detected in Djougou (15.39%; 95% CI: 2.71–46.34) and Matéri (14.29%; 95% CI: 0.75–57.99). However, there was no significant difference in the seroprevalence obtained for the different districts (Table 2).

The effect of sex on CCHFV seropositivity in cattle showed higher prevalence in female cattle (70.59%; 95% CI: 52.33–84.29) compared to males (29.41%; 95% CI: 15.71–47.67) with a significant difference. Also, the results indicated that seropositivity was significantly associated with age, as seropositive cattle clustered in the [0; →[ years age range. Seroprevalences were significantly higher in the age range of [5; 10[ years (52.94%; 95% CI: 35.40–69.84; *p* < 0.05) followed by [10; →[ years (32.35%; 95% CI: 17.98–50.63; *p* < 0.05). Herd movement was another significant factor associated with seropositivity, as the highest seroprevalence was detected in nomadic cattle (55.88%; 95%CI: 38.09–72.28), followed by semi-nomadic herds (32.35%; 95%CI: 17.98–50.63). Sedentary herds had the lowest seroprevalence (OR = 0.11; *p* = 0.000; 95% CI: 0.02–0.41), suggesting minimal exposure to infection. Overall, CCHFV seropositivity was detected in all three agroecological zones of Benin for cattle, with the highest seroprevalence obtained in the West Atacora Zone IV (67.65%; 95% CI: 49.37–82.03), followed by the Central Benin Cotton Zone V (20.59%; 95% CI: 9.34–38.41). The lowest seroprevalence was reported in the North Benin Cotton Zone II (11.76%; 95% CI: 3.84–28.39) with a significant difference in the West Atacora zone IV (Table 3).

## 4. Discussion

Crimean–Congo hemorrhagic fever virus is a tick-borne viral pathogen responsible for emerging infectious diseases with cases and outbreaks reported in Africa [26]. It poses a significant threat to public health due to its extensive geographical distribution [27]. Although CCHF is asymptomatic in wild and domestic animals, these animals serve as sentinel species for disease surveillance and indicators of the virus’s presence in specific regions [14,28]. However, understanding of the virus’s transmission dynamics and distribution remains limited due to insufficient epidemiological data on CCHF prevalence in animals, humans and ticks. This study provides evidence of CCHFV circulation in Benin, with the detection of virus-specific antibodies in wild and domestic animals. Results showed a CCHFV seroprevalence of 3.7% (squirrels), 5.88% (hares) and 1.19% (giant rats). Thus, this assessment of the presence of CCHFV in wild animals showed a low prevalence of anti-CCHFV antibodies. Although these rates are low, they indicate natural exposure of these wildlife species to the virus in Benin. Small mammals such as rodents are considered suitable secondary hosts for ticks, potentially playing a role in the transmission of the infection through the tick vector [29]. Moreover, rodents spend a great deal of time foraging in leaf litter and brush where immature ticks are found. Thus, they serve as excellent hosts for the developmental stages of many tick species [30]. Also, the results of experimental studies have shown that hares and squirrels are important hosts for ticks and probably contribute to the amplification and transmission of CCHFV [14], while [31] reported that ticks collected from wildlife play an important role in the epidemiology of tick-borne viral diseases.

Seroepidemiological reports on viruses have detected anti-CCHFV antibodies in wild animals, such as multimammate rats, genet cats, birds (Senegal) and hedgehogs (Nigeria and Sudan) [3]. Additionally, CCHFV seroprevalence has been reported in a variety of species including hares, hedgehogs, warthogs, foxes, zebras and African buffaloes [15,32] Furthermore, CCHFV strains have been identified in other wild animals belonging to the Aves, Mammalia and Reptilia classes [10]. However, three CCHFV isolates were reported in hares and could play an important role in virus dynamics [14,33], thus showing the importance of wild animals in the epidemiology of CCHFV. Several rodent species including hares are known to constitute a significant source of animal protein in Benin [34]. Although anti-CCHFV antibodies were detected in wild animals in this study, they showed a low seroprevalence, which suggests a low dynamic infection from wildlife in the studied areas.

Serological screening also detected anti-CCHFV antibodies in cattle and horses, with an overall seroprevalence of 41.98% and 16.67% in northern and southern Benin, respectively. This result can be explained through the fact that *Hyalomma* species, the predominant vector of CCHFV, is one of the tick species that infests cattle and is distributed in all districts of Benin [35]. *Hyalomma* species have also been found in horses in Cameroon and Ethiopia [36,37], while serological studies conducted in Senegal reported a CCHFV seroprevalence of 70.31% in horses [37], thus showing that horses may act as reservoirs and facilitate virus transmission to humans. In addition, similar seroprevalence rates in cattle have been reported in countries with which Benin shares international borders and other countries in the West African sub-region, including Nigeria (24.0%, 30.43%), Niger (46.04%), Ivory Coast (53.57%), Mali (65.67%), Senegal (57.14%) and Mauritania (68.65%) [38,39,40,41,42,43]. Most West African countries are interconnected through transhumance and livestock trade, which likely facilitate the spread of ticks and CCHFV across international borders, thus emphasizing the importance of these practices in the epidemiology of CCHF in the sub-region. These movements of pastoralists/livestock traders and their animals can potentially influence the dynamics of introduction of CCHFV into countries where outbreaks had not been previously reported.

The high seroprevalence of CCHFV observed in older cattle may be attributed to prolonged exposure to infected ticks. Serological studies of CCHFV infections in Malawi and Gambia reported high seroprevalence of the virus in older cattle and also in female cattle [19,44]. According to [6], infected cattle, although asymptomatic, serve as amplifying hosts and can potentially transmit the virus to humans through direct contact or vector bites. Therefore, occupationally exposed persons such as herders, veterinarians and abattoir workers are at high risk, highlighting the need for awareness campaigns and preventive measures. The detection of anti-CCHFV antibodies in both cattle and horses in the present study underscores the risk of zoonotic transmission and the importance of continuous monitoring of domestic animals in endemic areas. Although no human cases of CCHF have been reported in Benin, this may reflect diagnostic limitations or underreporting. Moreover, surveillance of zoonotic diseases in livestock is crucial for identifying high-risk areas and preventing outbreaks. Efforts should therefore be focused on developing strategies to control arthropod-borne viruses such as CCHFV in Benin, with an emphasis on early detection and cross-border collaboration with neighboring countries to mitigate their spread. It is noteworthy that over the past decade, more than 500 cases of CCHF have been reported in Africa, resulting in hundreds of deaths [45]. While our study has limitations due to the sample size used, particularly with wildlife species, further studies are essential to investigate the endemicity of CCHFV in humans in Benin and to identify effective measures for disease prevention and control.

## 5. Conclusions

This study confirms the presence of CCHFV in wild and domestic animals in Benin, emphasizing the importance of seroepidemiological surveys in identifying high-risk areas for CCHFV transmission. Furthermore, the detection of CCHFV-specific antibodies in these animals indicates active virus circulation and shows that CCHFV constitutes a significant public health threat in Benin. This underscores the need for technical capacity building among animal and human health stakeholders to protect public health, especially in the absence of vaccines to prevent disease occurrence in both animals and humans. Establishing an early detection and early warning system is crucial to mitigating the risks of CCHF outbreaks and enhancing disease control efforts. Our findings reinforce the role of cattle as potential indicators and sentinels of CCHFV in endemic regions. Further research is essential to better understand the dynamics of vector–host–environment interactions, as well as the relationship between infection dynamics and transhumant cattle movement. These insights will contribute to the development of targeted strategies for CCHFV surveillance, prevention and control in Benin.

## Figures and Tables

**Table 1:** Seroprevalence of Crimean–Congo hemorrhagic fever in wild animals across different districts of Benin.

Variable	* n/N	Seroprevalence % ± 95% CI	Odds Ratio ± 95% CI	*p*-Value
Overall	3/254	1.18 (0.31–3.70)		
Species				
Giant rat	1/84	1.19 (0.06–7.38)	REF	-
Squirrel	1/27	3.7 (0.19–20.89)	0.32 (0.004; 25.53)	0.17
Hare	1/17	5.88 (0.31–30.76)	0.20 (0.002; 16.07)	0.09
Grasscutter	0/49	0 (0.00–9.06)	-	0.50
Crow	0/3	0 (0.00–69.00)	-	0.50
Bat	0/30	0 (0.00–14.13)	-	0.50
Birds	0/21	0 (0.00–19.24)	-	0.50
Antelope	0/5	0 (0.00–53.71)	-	0.50
Monkey	0/9	0 (0.00–37.12)	-	0.50
Cattle egret	0/9	0 (0.00–37.12)	-	0.50
District/wild animal				
Aplahoué	2/56	3.57 (0.62–13.38)	REF	-
Matéri	1/4	25 (1.32–78.06)	8.33 (0.12–209.41)	0.04
Allada	0/65	0 (0.00–6.95)	-	0.06
Adjarra	0/10	0 (0.00–34.45)	-	0.26
Kpomassè	0/36	0 (0.00–12.01)	-	0.11
Djidja	0/41	0 (0.00–10.67)	-	0.01
Adja-Ouèrè	0/4	0 (0.00–60.42)	-	0.34
Tanguiéta	0/11	0 (0.00–32.15)	-	0.25
Banikoara	0/5	0 (0.00–53.71)	-	0.33
Djougous	0/9	0 (0.00–37.12)	-	0.27
Natitingou	0/9	0 (0.00–37.12)	-	0.27
Parakou	0/3	0 (0.00–69.00)	-	0.36
Pobè	0/1	0 (0.00–94.54)	-	0.36

* n = Number of samples positive for CCHFV antibodies; N = total number of samples tested; CI = confidence interval; REF = reference.

**Table 2:** Seroprevalence of Crimean–Congo hemorrhagic fever in domestic animals across different districts of Benin.

Variable	* n/N	Seroprevalence % ± 95% CI	Odds Ratio ± 95% CI	*p*-Value
Overall	38/112	33.93 (25.42–43.56)	-	-
Species/domestic animal				
Cattle	34/81	41.98 (31.26–53.46)	REF	-
Horse	4/24	16.67 (5.48–38.19)	0.28 (0.06–0.94)	0.013
Pigeon	0/7	0 (0.00–43.91)	0 (0.00–1.04)	0.016
District/cattle				
Tanguieta	5/16	31.25 (12.13–58.52)	REF	-
Djougou	7/14	50.00 (26.80–73.20)	0.47 (0.08–2.53)	0.149
Matéri	11/17	64.71 (38.62–84.74)	0.26 (0.05–1.29)	0.031
Gogounou	4/9	44.44 (15.34–77.35)	0.58 (0.08–4.30)	0.254
Aplahoué	5/13	38.46 (15.14–67.72)	0.74 (0.12–4.44)	0.339
Djidja	2/12	16.67 (2.94–49.12)	2.21 (0.28–28.21)	0.197
District/horse				
Tanguieta	1/4	25.00 (1.32–78.06)	REF	-
Djougou	2/13	15.39 (2.71–46.34)	1.76 (0.02–46.78)	0.333
Matéri	1/7	14.29 (0.75–57.99)	1.87 (0.02–182.41)	0.334

* n = Number of samples positive for CCHFV; antibodies; N = total number of samples tested; CI = confidence interval; REF = reference.

**Table 3:** Seroprevalence and associated risk factors for CCHF in cattle.

Risk Factors		Cattle (34/81)			
		* n/N	% ± 95% CI	Odds Ratio ± 95% CI	*p*-Value
Sex	F	24/34	70.59 (52.33–84.29)	REF	-
	M	10/34	29.41 (15.71–47.67)	0.18 (0.05–0.55)	0.00
Age	[0–5[	5/34	14.71 (5.54–31.83)	REF	-
	[5–10[	18/34	52.94 (35.40–69.84)	6.33 (1.83–26.11)	0.00
	[10–→[	11/34	32.35 (17.98–50.63)	2.73 (0.74–11.53)	0.049
Transhumance	Nomadism	19/34	55.88 (38.09–72.28)	REF	-
	Sedentary	4/34	11.76 (3.84–28.39)	0.11 (0.02–0.41)	0.00
	Semi-nomadism	11/34	32.35 (17.98–50.63)	0.38 (0.13–1.13)	0.03
Agroecological zone	North Benin Cotton Zone II	4/34	11.76 (3.84–28.39)	REF	-
	West Atacora Zone IV	23/34	67.65 (49.37–82.03)	14.91 (3.93–73.07)	0.00
	- Central Benin Cotton Zone V	7/34	20.59 (9.34–38.41)	1.93 (0.43–9.99)	0.17

* n = Number positive for CCHFV antibodies; N = total number of samples tested; CI = confidence interval; M = male; F = female; REF = reference.

## Data Availability

Data is available upon the request to the corresponding author.
